# Evolution of Interferon-Based Therapy for Chronic Hepatitis C

**DOI:** 10.1155/2010/140953

**Published:** 2010-10-10

**Authors:** Chun-Hao Chen, Ming-Lung Yu

**Affiliations:** ^1^Digestive Division, Department of Internal Medicine, Kaohsiung Municipal United Hospital, Kaohsiung 804, Taiwan; ^2^Department of Internal Medicine, Kaohsiung Municipal Ta-Tung Hospital, Kaohsiung Medical University Hospital, Kaohsiung Medical University, Kaohsiung 807, Taiwan

## Abstract

Since 1986, interferon-alfa (IFN-*α*) monotherapy has been administered for patients with chronic hepatitis C (CHC). However, sustained response rate is only about 8% to 9%. Subsequent introduction of ribavirin in combination with IFN-*α* was a major breakthrough in the treatment of CHC. Sustained virological responses (SVRs) rate is about 30% in hepatitis C virus genotype 1 (HCV-1) patients, and is about 65% in HCV-2 or -3 patients. After 2000, pegylated interferon (PegIFN) much improved the rates of SVR. Presently, PegIFN-*α*-ribavirin combination therapy has been current standard of care for patients infected with HCV. In patients with HCV-1, treatment for 48 weeks is optimal, but 24 weeks of treatment is sufficient in HCV-2 or -3 infected patients. Clinical factors have been identified as predictors for the efficacy of the IFN-based therapy. The baseline factor most strongly predictive of an SVR is the presence of HCV-2 or -3 infections. Rapid virological response (RVR) is the single best predictor of an SVR to PegIFN-ribavirin therapy. If patients can't achieve a RVR but achieve a complete early virological response (cEVR), treatment with current standard of care can provide more than 90% SVR rate. HCV-1 patients who do not achieve an EVR should discontinue the therapy. Recent advances of protease inhibitor may contribute the development of a novel triple combination therapy.

## 1. Introduction

Interferon-alfa (IFN-*α*) monotherapy has been found with normalization of alanine aminotransferase (ALT) levels in a few patients diagnosed as non-A, non-B hepatitis even before hepatitis C virus (HCV) was identified as the chief etiologic agent in this diagnosis [1]. In 1989, the first cases of successful treatment of documented chronic hepatitis C (CHC) with IFN-*α* monotherapy were reported, but relapse after the cessation of treatment was common [2, 3]. The introduction of combination therapy with IFN-*α* and ribavirin has markedly improved treatment response. Nevertheless, less than one-half of patients with CHC were able to experience a favorable response to the combination therapy [4–6]. Since 2000, the attachment of inert polyethylene glycol to conventional IFN-*α*, pegylated IFN-*α* (PegIFN-*α*), reduced degradation and clearance, prolonging the half-life of IFN and permitting less frequent, weekly dosing while maintaining higher sustained IFN levels (compared with 3 times weekly for conventional IFN). Now, PegIFN-*α*-ribavirin combination treatment has been recommended for all patients infected with HCV. For patients infected with HCV genotype 1 (HCV-1), the recommended treatment duration is 48 weeks, whereas for patients infected with HCV-2 or HCV-3, the recommended treatment duration is 24 weeks [7].

## 2. Approved Agents for Treatment of Hepatitis C

### 2.1. IFN-*α*

 IFNs are natural cellular proteins with a variety of actions. There are two distinct but complementary mechanisms for the antiviral effects of IFN-*α*: (a) induction of a non-virus-specific antiviral state in infected cells, resulting in direct inhibition of viral replication, and (b) immunomodulatory effects that enhance the host's specific antiviral immune responses and may accelerate the death of infected cells [8]. A number of different IFNs have been used [9]. The U.S. Food and Drug Administration (FDA) has approved 3 IFN preparations for treatment of HCV: (a) 3 million units (MUs) IFN-*α*-2a 3 times weekly; (b) 3 MUs of IFN-*α*-2b 3 times weekly; and (c) 9 *μ*gs of IFN alfacon-1 twice weekly, or 15 *μ*g 3 times weekly in nonresponders [10].

### 2.2. Peginterferon (PegIFN)

 PegIFN is a product of pegylation to conventional IFN (the attachment of inert polyethylene glycol (PEG) polymers to a therapeutic protein such as IFN). The larger molecular size of the compound results in a longer half-life due to reduced clearance, while retaining biological activity, and allows more convenient once-weekly dosing. Two PegIFNs [11, 12] were studied: (a) PegIFN-*α*-2a, a 40 kDa branched molecule with a terminal half-life of 80 hours (range: 50–140 hours) and a mean clearance of 22 mL/hr·kg administered at a fixed 180 *μ*g per week and (b) PegIFN-*α*-2b, a 12 kDa linear molecule with a mean terminal half-life of 40 hours (range: 22–60 hours) and a mean clearance of 94 mL/hr *·* kg, administered on the basis of weight (1.5 *μ*g/kg/week). Maximal serum concentrations (*C*_max_) occur between 15 and 44 hours post dose and are sustained for up to 48–72 hours. These two PegIFNs much improved the rates of SVR in comparison with their nonpegylated counterparts [11, 12].

### 2.3. Ribavirin

 Ribavirin (1-*β*-D-ribofuranosyl-1,2,4-triazole-3-carboxamide) is an oral purine nucleoside analogue with broad activity against viral pathogens [13]. Clearance of ribavirin is markedly reduced with renal insufficiency [14]. The mechanism of action of ribavirin in CHC remains controversial. Among the suggested, but not proven, roles of ribavirin in the treatment of CHC are an immunologic modulation through switching the T-cell phenotype from type 2 to type 1; inhibition of host inosine monophosphate dehydrogenase activity; depletion of intracellular guanosine triphosphate pools; induction of mutational catastrophe; or a moderate, transient, early direct antiviral effect [15]. Ribavirin may lead to rapid and lethal mutation of virions or depletion of intracellular guanosine triphosphate, which is necessary for viral RNA synthesis [16]. Additionally, ribavirin may act synergistically with IFN by upregulating the activity of double-stranded RNA-activated protein kinase and enhances the action of interferon-alfa against hepatitis C virus [17].

 The most interesting clinical observation is that ribavirin monotherapy had a minimal effect on HCV viremia, despite the fact that serum ALT levels were reduced significantly in a considerable proportion of patients with chronic HCV infection [18]. However, the combination of ribavirin and IFN provides a clinically synergistic anti-HCV effect. Hence it was proposed that ribavirin may exert its effect on the host immune response. Several studies on virus-specific T-cell reactivity in association with IFN treatment have found increased numbers of patients with CHC with demonstrable HCV-specific Th responses either during treatment or after a sustained therapeutic response. These findings raise the possibility that enhancement of HCV-specific T-cell reactivity may be one mechanism for successful antiviral treatment. HCV-specific T-cell reactivity was uncommon at baseline but increased markedly during antiviral therapy, peaking around treatment weeks 4–8 [19]. The main difference in T-cell reactivity of patients treated with IFN-ribavirin was a significant decrease of the expression of IFN-*ϒ*, whereas INF-*ϒ* expression was similar to that in patients receiving IFN monotherapy. The greater efficacy of ribavirin may exert an anti-inflammatory effect and may help reducing IFN-*γ*-driven T-cell activation and liver damage [20].

## 3. Assessment of Treatment Response for Hepatitis C

 In earlier studies, the primary end point for HCV therapies was a biochemical response, defined as the normalization of ALT levels [2, 3]. The introduction of virologic assays to detect HCV RNA further allows the assessment of a virologic response, defined as polymerase chain reaction- (PCR-) seronegative (≦50 IU/mL, or 100 copies/mL) for HCV RNA. Histological response has been assessed in some clinical studies, but there is little indication for posttreatment biopsy in clinical practice.

 Four on-treatment and three patterns of off-treatment virological responses to antiviral therapy for hepatitis C have emerged over the past decade [21–23]. They include the following:

1. *rapid virologic response (RVR):* PCR-seronegative of HCV RNA at week 4;
2. *early virologic response (EVR):* there are two kinds stratifications of EVR:
  - complete EVR (cEVR): PCR-seronegative of HCV RNA at week 12;
  - partial EVR (pEVR): decrease of HCV RNA by >2 log from baseline values at week 12;
3. * end-of-treatment virologic response (ETVR):* PCR-seronegative of HCV RNA at the end of therapy;
4. * virologic breakthrough:* HCV RNA reappearance in serum while still on treatment;
5. *sustained virologic response (SVR): *PCR-seronegative of HCV RNA 6 months after completing therapy;
6. *virologic Relapse: *PCR-seronegative of HCV RNA at the end of therapy, with return of circulating virus after completion of therapy;
7. *nonresponders:* persistently seropositive for HCV RNA throughout treatment.

More than 97% of patients with SVR remain nonviremic by PCR for the subsequent 5–14 years [24, 25]. These patients are regarded as having a high probability of a durable biochemical, virologic, and histological response [26].

## 4. Evolution of IFN-Based Therapy for Chronic Hepatitis C

### 4.1. IFN-*α* Monotherapy

Until the 1990s, the only therapy of proven benefit for patients with CHC was IFN-*α*. Initially, a 6-month course of 3 weekly injections of 3 MUs of IFN-*α* was approved for treatment of CHC, and a biochemical response, defined as the normalization of ALT levels, was assigned as the primary end point [2, 3]. IFN-*α* monotherapy suppresses serum HCV RNA to undetectable levels and normalizes the ALT level in 25% to 40% of CHC patients, usually within the first 2-3 months of treatment. However, these initial responses to IFN-*α* monotherapy are usually transient, and sustained response is documented in only about 8% to 9% of patients [27].

When virologic assays for detection of HCV RNA became available, the virological response rates were observed to be lower than those reported with biochemical end points. In the meta-analysis of IFN-*α* monotherapy [28], normalization of ALT levels at the end of treatment and 6 months after stopping treatment was seen in 47% and 23% of treated patients, respectively. ETVR and SVR, however, were observed in only 29% and 8% of treated patients, respectively. Improvement of efficacy on CHC could be achieved with higher doses and/or a longer duration of IFN-*α* monotherapy. A doubling of the duration of therapy to 12 months increased the frequency of SVRs to approximately 20%. The best efficacy/risk ratio was in favor of 3 MUs of IFN-*α* 3 times weekly for at least 12 months in treatment-naïve patients with CHC [27].

### 4.2. IFN-*α* and Ribavirin Combination Therapy

 The introduction of ribavirin in combination with IFN-*α* was a major breakthrough in the treatment of CHC. Even though ribavirin monotherapy was shown to be ineffective [18], the rate of SVRs was 43% and 6% for the IFN-*α*-2a with and without ribavirin combination [4], respectively, and 36% and 18% for the IFN-*α*-2b with and without ribavirin combination [29]. A meta-analysis in 1995 showed that the SVR rate was significantly higher for IFN-ribavirin combination therapy than for IFN or ribavirin monotherapy (odds ratio [OR]: IFN-ribavirin versus IFN = 9.8; 95% confidence interval [CI] = l.9–50) [30]. 

 Several landmark studies then followed and consistently demonstrated the dramatically improved responses to combination therapy, especially for HCV-2 or HCV-3 patients. In 1998, two multicenter randomized controlled trials (RCTs) (one U.S. study and one international study) totaling 1,744 previously untreated patients with compensated CHC compared 24- and 48-week drug regimens of IFN-*α*-2b monotherapy (3 MUs 3 times weekly) with those of IFN-*α*-2b and ribavirin (1.000 mg/day or 1.200 mg/day for patients weighing <75 kg or >75 kg, resp.) combination therapy followed by 24 weeks of off-therapy followup [5, 6]. The overall SVR rates for 24 and 48 weeks of therapy were 33% and 41%, respectively, for patients receiving IFN-*α*-2b-ribavirin, compared with SVR rates of 6% at 24 weeks and 16% at 48 weeks IFN-*α*-2b monotherapy. In addition to definitively showing the benefit of combination therapy over IFN alone, these studies made several other important clinical points. First, a striking reduction in hepatic inflammation was seen in sustained virological responders. Second, the likelihood of response to treatment was related to pretreatment virus level and genotype. SVRs to 48 or 24 weeks of combination therapy occurred in 29% and 17% of HCV-1 patients, respectively, and in 65% and 66% of HCV-2 or HCV-3 patients. The two studies reinforced the importance of longer duration therapy for 48 weeks in patients with HCV-1 infection. Similarly, SVRs to 48 or 24 weeks of combination therapy occurred in 38% and 27% of patients with pretreatment HCV RNA levels of greater than 2 × 10^6^ copies/mL, respectively, but the SVR rates were no different for those with lower levels (45% and 43%, resp.). A systematic review in 2001 included data from 15 trials in which patients received IFN-*α* monotherapy or IFN-*α*-ribavirin combination therapy. In comparison with IFN-*α* monotherapy, combination therapy reduced the nonresponse rate (absence of SVR) by 26% in treatment-naïve patients (relative risk = 0.74; 95% CI = 0.70–0.78). Morbidity and mortality showed a nonsignificant trend during treatment in favor of combination therapy.

 In 1998, the FDA approved the combination of IFN-*α* and ribavirin for patients with chronic HCV infection. In 1999, the EASL International Consensus Conference on Hepatitis C recommended that, for patients with CHC who have not been previously treated, (a) standard therapy should consist of IFN-*α* and ribavirin in combination for 24 weeks and that (b) treatment should be extended to 48 weeks in patients with both HCV-1 and HCV RNA levels greater than 2 × 10^6^ copies/mL [31].

### 4.3. PegIFN-*α* Monotherapy

 Four RCTs compared the efficacy and safety of once-weekly PegIFN-*α* monotherapy compared with IFN-*α* monotherapy three times per week for the treatment of chronic HCV infection in treatment-naïve patients [11, 12, 32, 33]. The initial studies of PegIFN-*α* evaluated the dose-ranging efficacy of monotherapy. The recommended dose of PegIFN-*α*-2a monotherapy, administered fixed at 180 *μ*g/week for 48 weeks, achieved higher SVR rates compared with IFN-*α*-2a monotherapy (30% to 39% versus 8% to 19%) [12, 32, 33]; the PegIFN-*α*-2b monotherapy, administered according to body weight at 1.5 *μ*g/kg/week for 48 weeks, achieved an SVR rate of 23%, compared to 12% with IFN-*α*-2b monotherapy [11].

 Of note, Heathcote et al. [32] conducted the first substantive prospective study confined to patients with compensated cirrhosis or advanced fibrosis. Cirrhosis has been a poor predictor of responsiveness and is associated with a high risk of leucopenia and thrombocytopenia [5, 6]. This study, however, showed that PegIFN monotherapy was both well tolerated and effective in cirrhotic CHC patients, with an SVR rate of 30%.

 PegIFN monotherapy has been recommended for patients with contraindications to ribavirin, such as those with renal insufficiency, hemoglobinopathies, and ischemic cardiovascular disease. Some clinical trials have been reported to date in these populations [34, 35]. For patients with contraindications to ribavirin but who have indications for antiviral therapy, PegIFN represents the best option of treatment.

### 4.4. PegIFN-*α* and Ribavirin Combination Therapy

 The results of PegIFN-*α* monotherapy encouraged more clinical trials to go on and anticipation that combination therapy with PegIFN-*α* and ribavirin would be even more effective. The earlier two large RCTs were applied with fixed durations of 48 weeks [36, 37]. In these trials, PegIFN-*α*-2b was dosed by weight (1.5 *μ*g/kg was FDA approved) and coupled with 800 mg of ribavirin; PegIFN-*α*-2a was given at a fixed dose of 180 *μ*g along with a weight-adjusted, higher dose of ribavirin (1.000 mg/day or 1.200 mg/day for patients weighing <75 kg or >75 kg, resp.). The overall response rate in clinical trials was 54% to 56%. These trials demonstrated that higher SVR rates could be achieved with the combination of PegIFN-*α* weekly plus oral ribavirin given twice daily than with the combination of IFN-*α* given 3 times weekly plus ribavirin or than with PegIFN-*α* monotherapy. 

 The issue of influence of ribavirin dose by body weight on the response rate was first addressed. In the PegIFN-*α*-2b study, a post hoc analysis demonstrated that an SVR of 61% was achieved in the subgroup whose daily dose of ribavirin exceeded 10.6 mg/kg. Logistic regression analyses observed that the response rates generally increased as ribavirin dose increased up to about 13 mg/kg/day. Actually, the optimal ribavirin dose has not been defined. Some studies highlighted the potential importance of higher doses of ribavirin [38, 39]. The first 4 weeks of weight-based ribavirin exposure (>13 mg/kg/day) have been associated with the achievement of an RVR [40]. In non-RVR patients, one post hoc analysis showed that providing and maintaining optimal dose of ribavirin within 12 weeks of treatment was pivotal for the attainment of a cEVR [41]. Patients with a cEVR in this study received a ribavirin dose of 16.3 mg/kg/day. Moreover, a higher weight-based dose of ribavirin (15.2 mg/kilogram/day) was associated with a lower relapse rate and higher SVR rate [42].

 Later, the optimal treatment duration and ribavirin dose were investigated in a multicenter RCT in which all CHC patients received PegIFN-*α*-2a at a dose of 180 *μ*g, while patients in the four arms received either 24 or 48 weeks of ribavirin at a dose of 800 mg or at the higher, weight-based doses of 1.000 or 1.200 mg daily [43]. In the subsequent registration trial, a high frequency of SVRs occurred in patients with HCV-2 or HCV-3, regardless of the regimen (79% to 84%), but optimal frequencies of SVRs in HCV-1 (52%) required longer duration and full-dose ribavirin, independent of the level of HCV RNA. In patients with HCV-1 with a low viral load (<2 × 10^6^ copies/mL, or 800.000 IU/mL), the SVR was highest in those who had received the higher ribavirin dose and who were treated for 48 weeks (61%). This regimen was also optimal for patients with HCV-1 and a high viral load (SVR rate: 46%). In contrast, in patients with HCV-2 or HCV-3, regardless of the pretreatment viral load, no differences were detected with the 4 treatment regimens. Another single-arm, open-label, historical-control study of 24 weeks of treatment with PegIFN-*α*-2b plus ribavirin limited to patients with HCV-2 or HCV-3 demonstrated that 24 weeks of treatment was sufficient in HCV-2- or HCV-3-infected patients, with an overall SVR rate of 81% [44]. This study supports the current recommendations that patients with HCV-1 require 48 weeks of PegIFN-*α* therapy with higher doses of ribavirin, while patients with HCV-2 or HCV-3 can be treated for only 24 weeks and with only 800 mg daily of ribavirin [7, 45].

So far, there are 3 RCTs to compare the rates of SVR of these two PegIFNs. One RCT showed no significant difference between the two available peginterferon-ribavirin regimens in patients infected with HCV genotype 1 [46]. Two RCTs showed that SVR rates were significantly greater in HCV patients treated with PegIFN-*α*-2a than patients treated with PegIFN-*α*-2b [12, 47, 48]. One recent meta-analysis showed that peginterferon alpfa-2a significantly increased the number of patients who achieved a sustained virological response (SVR) versus peginterferon alfa-2b (47% versus 41%; risk ratio: 1.11; 95% confidence interval: 1.04–1.19; *P* = .004 (eight trials)) [49]. 

### 4.5. Contraindication and Adverse Events of IFN-Ribavirin and Management

 Contraindications and adverse events of IFN-ribavirin therapy are listed in Table 1. Physicians should look specifically for contraindications to antiviral therapy and assess both therapeutic risk and benefit. Ribavirin is contraindicated in pregnancy, necessitating strict precautions and contraception in women of childbearing age and their sexual partners and in HCV-infected men with female partners of childbearing age. Flu-like side effects of IFN can be managed with acetaminophen or nonsteroidal anti-inflammatory drugs; antidepressants and hypnotics can be used for depression and insomnia, respectively. For management of neutropenia, dose reduction suffices; the addition of granulocyte colony-stimulating factor is generally not recommended, although it may be considered in individual cases of severe neutropenia. Treatment with ribavirin should be avoided in patients with ischemic cardiovascular and cerebrovascular disease and in patients with renal insufficiency. If anemia occurs, options include ribavirin dose reduction or the addition of erythropoietin. Patients with decompensated cirrhosis are at high risk of adverse events and relatively contraindicated to IFN-ribavirin. 

 Patients receiving combination therapy had an increased risk for requiring medication dose reduction (RR = 2.44; 95% CI = 1.58–3.75) or discontinuation (RR = 1.28; 95% CI = 1.07–1.52) compared with those receiving IFN monotherapy [50]. The rates of IFN dose reduction and discontinuation were similar among subjects receiving PegIFN and conventional IFN [11, 12].

## 5. Factors Associated with Treatment Efficacy

With the great progress in the management of CHC, clinical factors have been identified as predictors for the efficacy of the IFN-based therapy. They could be divided into two major categories: baseline and on-treatment predictors (Table 2).

### 5.1. Baseline Predictors of Response to IFN-Based Therapy

#### 5.1.1. Virologic Factors

 The pretreatment variable most strongly predictive of an SVR is the presence of HCV-2 or HCV-3 infection [51], whether with conventional IFNs or PegIFNs, alone or in combination with ribavirin [5, 6, 36, 37]. On the basis of variations in the nucleotide sequence of HCV, six genotypes (numbered 1–6) and more than 50 subtypes (identified by lowercase letters, e.g., 1a and 1b) have been identified [52]. Why HCV-1 is harder to treat than other HCV genotypes is not yet fully understood. Several studies demonstrated that there exists a genotype-specific difference of viral kinetics [23, 53]. The turnover of hepatocytes infected with HCV-1 is slower than that of hepatocytes infected with other HCV genotypes after initiation of IFN-based therapy [53, 54], implying that HCV-1 might be more resistant to antiviral therapy. Under the current recommendation [7], SVR rates were 42% to 60% for HCV-1 infection with a 48-week PegIFN-ribavirin treatment, compared with 76% to 95% for HCV-2 or HCV-3 infections with a 24-week regimen [23, 36, 37, 43, 44, 55, 56]. Patients with HCV-4, which is common in Egypt, are intermediate in responsiveness to therapy between those infected with HCV-1 and HCV-2 or HCV-3, and it is suggested that they should be treated for a full 48 weeks with full-dose ribavirin, like patients with HCV-1 [4, 41]. There is insufficient experience to provide recommendations for the treatment of persons with HCV-5 and HCV-6 so far. Experienced providers need to make treatment judgments on a case-by-case basis. Since HCV genotype is the strongest predictor of responses to IFN-based therapy for CHC, it should be determined in all HCV-infected persons prior to treatment to determine the duration of therapy and the likelihood of response [7].

 Pretreatment HCV RNA level, even less important than HCV genotype, is a predictor of sustained response in IFN-based therapy [5, 6, 11, 37, 57]. A higher HCV RNA level predicts a lower response rate. The impact of HCV RNA level on the response to combination therapy was different between patients with different HCV genotype infections. High viral load (with a cutoff value of 200.000 copies/mL, or 40.000 IU/mL) influenced the response rate in patients with HCV-1 (41% versus 56%) but not those in patients with HCV-2 or HCV-3 (74% versus 81%) [36]. Under the circumstances of a determined HCV genotype for CHC patients, testing HCV RNA levels is beneficial and recommended for HCV-1 patients but seems variable for HCV-2 or HCV-3 patients [7].

 HCV viral quasispecies evolution is considered another key element determining treatment response [58]. Higher quasispecies complexity at baseline has been observed in nonresponders than in sustained virological responders [59]. An increasing number of mutations within the carboxyl terminal region of the HCV nonstructural 5A protein, named the IFN-sensitivity-determining region (ISDR), were correlated with treatment response in HCV-1-infected patients [60]. Patients infected with the so-called mutant type, defined by four or more amino acid substitutions in the ISDR, showed a more favorable response toward IFN-based therapy in Japan and Taiwan [60, 61]. However, these findings were not observed in a European study [62]. Additionally, a high degree (≥6) of sequence variation in the variable region 3 (V3) plus its upstream flanking region of NS5A (amino acid 2334–2379), referred to as IFN/RBV resistance-determining region (IRRDR), would be a useful marker for predicting SVR, whereas a less diverse (≤5) IRRDR sequence predicts non-SVR [63].

#### 5.1.2. Host Factors

 The presence of bridging fibrosis and cirrhosis has been reported as one of the most unfavorable predictors for IFN-based therapy [5, 6, 12, 51, 64, 65]. Patients with cirrhosis generally respond poorly to standard IFN monotherapy, with SVR rates of 5% to 20% [6, 32]. Responses are improved when conventional IFNs or PegIFNs are combined with ribavirin, resulting in SVR rates of 33% to 44% [6, 36, 37].

 A gender effect on response has been reported. Female sex was a predictor of SVR in studies of conventional IFN-based therapy [51], but not in the studies of PegIFN-ribavirin [11, 36, 43]. Younger patients (<40 years) had higher SVR rates with PegIFN-ribavirin [36, 37, 43]. Sustained responders were younger than nonresponders by an average of 5 years [66].

 Several studies have demonstrated that SVR rates are lower in patients with coexistent insulin resistance and/or hepatic steatosis or steatohepatitis [67, 68]. In HCV-1 patients treated with PegIFN-ribavirin, a lower SVR rate was observed in patients with insulin resistance (homeostasis model of assessment, HOMA-IR > 2) compared to those without insulin resistance [69, 70].

 CHC patients with body mass indexes >30 kg/m^2^ are more likely to be insulin-resistant, to have more advanced hepatic steatosis or steatohepatitis and fibrosis, and to experience a reduced response to combination therapy [71, 72]. Additionally, other possible mechanisms of the impact of obesity on the therapeutic response include the linear correlation of efficacy and body-weight-based doses of ribavirin (10.6–15 mg/kg/day) [37]. To encourage weight loss and exercise before treatment, which has been associated with a reduction in steatosis fibrosis scores, is the most direct approach for formulating more effective treatment regimens [73].

 Excessive alcohol use could reduce the likelihood of a response to therapy [74, 75]. To increase the efficacy of antiviral therapy, it has been suggested that abstinence be recommended before and during treatment for CHC [45].

 Racial differences in response to efficacy of IFN exist and have been one of the host factors. A lower response rate to IFN monotherapy was observed among African-American patients compared with White patients [17, 76]. A pool analysis of two clinical trials with IFN-ribavirin combination therapy demonstrated that SVRs were highest among Asians (61%), followed by Whites (39%), Hispanics (23%), and African-Americans (14%) [77]. Hispanics and African-Americans were less likely to respond to PegIFN-*α*-ribavirin compared to Whites [78]. In studies of Taiwanese CHC patients, the SVR rate was 23.7%, 37.1%, and 63.6% for a 24-week treatment of 3 MUs of IFN-*α* 3 times weekly alone, 6 MUs of 3 times weekly alone, and 3 MUs of 3 times weekly plus ribavirin, respectively [65, 79]. The SVR rate of HCV-1b patients to 24-week PegIFN-*α*-ribavirin was 48.9% to 65.8% and could be as high as 80% with a 48-week regimen in Taiwan [79, 80]. A relative lower body weight (67–70 kg) in Asian patients compared to U.S. patients (78–81 kg) may also play an important role [71]. 

The different ethnic response rates may reflect the important role of genetics. Host genetic variations are probably involved in the efficacy of IFN-based therapies for CHC [81]. Genetic polymorphisms of human leukocyte antigen, CC chemokine receptor 5, cytotoxic T lymphocyte antigen-4, interleukin-10, low molecular mass polypeptide 7, MxA, and transforming growth factor-*β*1 have been reported to have significant associations with responsiveness [82–89]. TNF-*α*-308 polymorphism was associated with SVRs to IFN-ribavirin in patients with HCV-1b infection and a high viral load [90]. These results reflect the important role of unique genetic predisposition, at least in part, in the response to IFN-based therapy for CHC. Recent advances in pharmacogenomics have demonstrated the potential applications of genetic single nucleotide polymorphism and expression patterns in determining treatment responsiveness in CHC [91, 92]. A recent candidate gene study showed that genetic variation in the *IL28B *gene, which encodes IFN-*λ*3, is associated with spontaneous HCV clearance [93]. Several genome-wide associated studies observed that IL28B single nucleotide polymorphisms played an important role in the treatment outcome of PegIFN-RBV for CHC [94–96]. A genome-wide association study in 2010 confirmed that IL28B genetic variation was the strongest genetic predictor in both natural and treatment-induced control of HCV. No SNP outside the IL28B/A locus reached genome-wide significance [97]. The increasing evidence for the role of IFN-*λ*3 for both spontaneous and treatment-induced control of HCV infection opens new avenues for prognosis and treatment of HCV infection. Individuals with HCV genotype 1 or 4 who carry the risk allele, particularly in homozygosis, will have a very low probability of natural or treatment-induced clearance. These individuals would be prime candidates for novel therapeutic strategies [97]. Half of the ethnic differences in response to interferon and ribavirin combination therapy can be explained by genetic polymorphism of IL28B [94].

 Because of the presumably shared routes of transmission, approximately one-fourth to one-third of all persons infected with HIV are coinfected with HCV [98]. Patients with HIV-HCV coinfection have been shown to respond less favorably to antiviral therapy than patients infected with HCV alone [98, 99]. Several RCTs recommended 48 weeks of PegIFN-ribavirin for HCV, regardless of HCV genotype, in HCV-HIV coinfected patients [100, 101].

 Dual infections of HCV and hepatitis B virus (HBV) are not uncommon and occur in up to 5% of the general population in HCV-endemic areas [102]. Combined chronic hepatitis B and C leads to more severe liver disease and an increased risk of HCV [103]. Although HBV-HCV dual infection was refractory to conventional IFN monotherapy [104], recent studies in Taiwan have demonstrated that conventional IFN-ribavirin combination therapy was effective in HCV clearance among HCV-dominant, HBV/HCV dually infected patients [105, 106]. Recently, a large, open-label, comparative, multicenter study confirmed the efficacy of PegIFN-ribavirin for patients with chronic HCV-HBV dual infection in Taiwan [107].

 Nonresponders are more resistant to retreatment with subsequent IFN-based therapy, compared to relapsers (OR = 3.912; 95% CI = 1.459–10.49) [108]. Retreatment with PegIFN-ribavirin could achieve an SVR rate of 47% to 60% for relapsers and 18% to 23% for nonresponders [109–112].

### 5.2. On-Treatment Predictors and Response-Guided Individualized Therapy

 During IFN-*α*-based therapy, HCV RNA levels generally fall in a biphasic manner [74]. The first rapid phase of viral suppression, from a few hours after the first IFN-*α* injection to the end of the first day, is related to an inhibition of viral replication by a direct, nonspecific action of IFN-*α*. This early initial decline in HCV RNA levels correlates poorly with the eventual response to IFN-based therapy [74, 113]. The second, slower phase of viral suppression, beginning on day 2 and gradually leading to seroclearance of HCV RNA, is possibly related to the gradual clearance of infected cells by the patient's immune system, while HCV replication is efficiently inhibited. This phase, less influenced by the dosage of IFN and HCV genotype, exhibits a good response to PegIFN and is an excellent marker of an SVR to the treatment [36, 54, 74].

 An RVR at week 4 could predict an SVR to IFN-ribavirin with a high degree of accuracy in both HCV-1 and HCV-2 patients, with positive predictive values of 78% and 92%, respectively [23]. Recent studies have demonstrated that an RVR is the single best predictor of an SVR to PegIFN-ribavirin for HCV-1 [114, 115] and HCV-2 or HCV-3 patients [23, 55, 56, 116]. For HCV-1 or HCV-4 patients with lower baseline viral loads and an RVR, an abbreviated 24-week regimen could achieve a comparable SVR rate with a standard 48-week regimen [115, 117, 118]. Selected patients with RVR might have their treatment courses abbreviated to 16 weeks if they are infected with HCV-2 or HCV-3 [23, 56]. But, the shortening of therapy duration for genotype 2/3 with RVR is still controversial [119]. Abbreviated regimens may be considered in patients with a low baseline viral load who achieve an RVR [120, 121].

 Among patients with an EVR, the likelihood of an SVR is only 72% [22]. However, as a negative predictor, non-EVR is even an more robust predictor. In cases without an EVR, the likelihood of an SVR is approximately 0% to 2% [122]. In Taiwan, the non-EVR is a significantly negative predictor in HCV-1 patients, but not in HCV-2 patients [23]. Thus it is recommended that HCV-1 patients who do not achieve an EVR at week 12 should discontinue the therapy beyond 12 weeks [22, 78]. Recently, stratification of early virological response (EVR) into complete EVR (cEVR) and partial EVR (pEVR) has been possible to further improve the prediction of an SVR and may allow for optimizing treatment duration in non-RVR patients [123]. Studies for HCV-1 non-RVR patients have demonstrated that the current recommended 48 weeks of treatment could achieve high SVR rates in patients with a cEVR but could lower rates of SVR in patients with a pEVR [124, 125]. The SVR rates would be more than 90% if patients could reach a cEVR with a standard regimen (48 weeks for HCV-1 or 24 weeks for HCV-2) [41]. For non-RVR patients, HCV viral loads <10^4^ IU/mL at week 4 provided an early and accurate prediction of who would or would not achieve a cEVR and following SVR [41]. In HCV-1-infected patients with a pEVR, the SVR rates were 10% and 21% only and the relapse rates were up to 83% and 63% in the 24-week and 48-week groups, respectively. The treatment responses were inadequate, even with a standard 48-week regimen in these patients [124, 125]. 

Based on these predictors associated with treatment efficacy, response-guided individualized therapy has become a major consideration for clinicians. It is desirable to expose CHC patients to the lowest doses and shortest durations of treatment, to reduce the likelihood of adverse events and to minimize costs, without compromising treatment efficacy. On the other hand, some difficult-to-treat patients have to receive longer and/or higher dose therapy to achieve responses. To date, HCV genotype, baseline viral load as well as on-treatment virological responses will provide information for individualized therapy decisionmaking for CHC patients in the near future [115, 126]. People who have an RVR may have a chance to abbreviate their treatment courses to avoid unnecessary costs and preventable drug side effects. In patients without an RVR treated with standard of care, the SVR rate would be more than 90% if cEVR could be accomplished. In patients with only a pEVR, it has been suggested to extend the treatment course to 72 weeks [124, 125, 127] or adhere to high-dose peginterferon plus ribavirin combination therapy [128]. In the future, additional therapy other than interferon-based treatment, such as protease inhibitors, might be anticipated in those difficult-to-treat patients. One would like to be able to evaluate whether a treatment response is likely as early as possible so that individualized strategies can be made or altered earlier before or during the treatment course. HCV viral loads <10^4^ IU/mL at week 4 provided an accurate prediction of cEVR and SVR in non-RVR patients [41].

Medical adherence is an important factor associated with response to IFN-ribavirin, especially among patients with HCV-1 infection. In a retrospective analysis of data collected in the large registration trials of IFN-ribavirin, SVRs have been reported to be more likely in patients who had taken at least 80% of all projected IFN injections and at least 80% of all projected ribavirin for at least 80% of the anticipated duration of treatment [39].

## 6. Protease Inhibitors and IFN-Based Therapy

Recent development of direct-acting antiviral agents, also named “specifically targeted antiviral therapy for hepatitis C” (STAT-C) compounds, to treat HCV has focused predominantly on inhibitors of the viral enzymes NS3/4A protease and the RNA-dependent RNA polymerase NS5B [129, 130]. NS5B polymerase inhibitors in general have a lower antiviral efficacy than protease inhibitors [130]. The administration of HCV NS3/4A protease inhibitors to patients with chronic HCV infections has demonstrated that they have dramatic antiviral effects and that compounds acting via this mechanism are likely to form a key component of future anti-HCV therapy [131]. Newer data have demonstrated promise for 2 protease inhibitors, SCH 503034 (boceprevir) and VX-950 (telaprevir), both of which appear to be able to improve sustained response while shortening duration of therapy [132]. Telaprevir (VX-950), the HCV protease inhibitor, is in the most advanced phase of clinical development [133]. A first case of sustained virological response (SVR) achieved in a patient with chronic hepatitis C by monotherapy with telaprevir without interferon therapy was reported [134]. Owing to a low genetic barrier, resistant variants emerge within a few days when used in monotherapy, thereby decreasing its efficacy. Consequently, telaprevir has been combined with pegylated-interferon and ribavirin in clinical trials. This triple combination is more effective but has a higher rate of adverse events (notably rash) than the standard of care, despite the shorter duration of therapy [133]. Results of the milestone studies PROVE 1 and 2 indicate that 12 weeks of telaprevir-based triple therapy is too short because of the high rate of relapse after treatment completion. However, 24 to 48 weeks of total therapy including 12 weeks of triple therapy with telaprevir in addition to standard treatment greatly improved SVR rates in treatment-naïve genotype 1 patients compared with the standard of care. PROVE 3 has shown that telaprevir is also highly effective in the treatment of prior nonresponders or relapsers infected with HCV genotype 1 [130, 135].

## Figures and Tables

**Table 1:** Contraindications and adverse effects of hepatitis C therapy.

Contraindications	

Absolute contraindications	Major, uncontrolled depressive illness; autoimmune hepatitis or other condition known to be exacerbated by interferon and ribavirin; untreated hyperthyroidism; pregnant or unwilling/unable to comply with adequate contraception; severe concurrent disease such as severe hypertension, heart failure, significant coronary artery disease, poorly controlled diabetes, obstructive pulmonary disease; under 3 years of age; known hypersensitivity to drugs used to treat HCV

Relative contraindications	Decompensated liver disease; solid organ transplantation (except liver); coexisting medical conditions: severe anemia (hemoglobin level < 100 g/L), neutropenia (neutrophil count < 0.75 × 10^9^/L), thrombocytopenia (platelet count < 40 × 10^9^/L), hemoglobinopathy, uncontrolled heart disease (angina, congestive heart failure, significant arrhythmias), cerebrovascular disease, advanced renal failure (creatinine clearance < 50 mL/min)

Adverse effects	

Interferon or peginterferon	Flu-like symptoms (fever, fatigue, myalgia and headaches); mild bone marrow suppression (especially, leucopenia and thrombocytopenia); gastrointestinal manifestation (anorexia, nausea, vomiting and diarrhea); emotional effects (depression, irritability, difficulty concentrating, memory disturbance and insomnia); dermatological manifestation (skin irritation, rash and alopecia); autoimmune disorders (especially thyroid dysfunction); weight loss; tinnitus and hearing loss; retinopathy (usually not clinically significant); hyperglycemia; seizures; renal function impairment; pneumonitis.

Ribavirin	Hemolytic anemia (dose dependent); cough and dyspnea; rash and pruritis; nausea; sinus disorders; teratogenicity.

**Table 2:** Factors associated with response to interferon-based therapy for hepatitis C.

Baseline
Virological factors
Hepatitis C virus genotype
Hepatitis C viral loads
Quasispecies

Host factors
Bridging fibrosis/cirrhosis
Gender
Age
Ethnicity
Insulin resistance
Obesity
Hepatic steatosis
Host genetics: genetic variation in IL28B
Coinfection with HIV
Nonresponse to previous interferon-based therapy

On-treatment

Rapid virological response (RVR) at week 4
Early virological response (EVR) at week 12
Complete EVR (cEVR) versus Partial EVR (pEVR)
Medical adherence
